# Differentiated SH-SY5Y cells exhibit neuronal features but lack synaptic maturity

**DOI:** 10.1038/s41420-026-03094-y

**Published:** 2026-04-14

**Authors:** Jana Leuenberger, Grischa Ott, Thomas Nevian, Benoît Zuber, Iman Rostami

**Affiliations:** 1 Institute of Anatomy, University of Bern, Bern, Switzerland; 2 Department of Physiology, University of Bern, Bern, Switzerland

**Keywords:** Cellular neuroscience, Extracellular signalling molecules

## Abstract

A vital question in neuroscience is whether and how efficiently cellular models may be differentiated into functional neuronal cells in culture. Despite the frequent use of the human neuroblastoma cell line SH-SY5Y, differentiation protocols vary extensively, with the most common being differentiation via the addition of retinoic acid and brain-derived neurotrophic factor. However, due to the lack of a reliable evaluation method, their adequacy as synaptic models remains unclear. Here, we investigate whether SH-SY5Y cells constitute a functional model for synaptic studies by phenotypically and ultrastructurally analyzing synaptogenesis in SH-SY5Y cells subjected to different differentiation protocols. Electron microscopy (EM) techniques, including conventional EM, cryo-EM, and cryo-electron tomography, were systematically applied to characterize synaptogenesis in SH-SY5Y cells. Further characterization was performed using immunostaining and functional assays, such as live exocytosis assays and whole-cell patch-clamp electrophysiology. Despite exhibiting some presynaptic-like features, differentiated SH-SY5Y cells do not form morphologically or functionally complete synapses under the conditions tested. Immunostaining results were consistent with previous findings, showing synaptic markers. However, functional investigations did not detect synaptic activity. High-throughput EM analyses revealed an absence of synaptic structures in these cells. Additionally, an alternative differentiation approach incorporating additional neurotrophic factors promoted the formation of presynaptic-like compartments containing synaptic vesicle-like vesicles (SVLVs). In contrast to typical synaptic vesicles, these SVLVs exhibited a pleomorphic size distribution and lacked connectors. These findings underscore the need for cautious interpretation of results derived from SH-SY5Y cells when investigating molecular synaptic architecture or function, as well as neurodegenerative diseases.

## Introduction

There are numerous neuronal cell models, among the most used ones are human neuroblastoma cell lines, such as SH-SY5Y, mouse neuroblastoma cell lines, such as NB41A, Neuro2a, and dopamine-containing hybrids (MN9D), used in neuronal differentiation, neurotoxicity, neurodegenerative diseases, and cancer [1–5]. Other widely used cell lines include rat-derived cells, such as catecholaminergic cells (PC12) [6–8], and human embryonic neuronal precursors (LUHMES) [9]. Furthermore, induced pluripotent stem cell (iPSC)-derived neurons are increasingly regarded as an alternative model for studying synapses due to their potential to differentiate into diverse neuronal subtypes and form structures that more closely resemble native neurons [10, 11]. However, the production of iPSC-derived neurons is resource-intensive, requiring specialized expertise and infrastructure, as well as expensive culture media and lengthy differentiation times. This and limited scalability make them less suitable for high-throughput applications compared to other models such as SH-SY5Y cells. As such, the choice of model often reflects a balance between physiological relevance and experimental feasibility. SH-SY5Y cells provide a faster and more cost-effective alternative for investigating neuronal characteristics. While their structural and functional complexity does not fully match that of primary neurons or animal models, their use facilitates scalable and ethically sustainable experimentation. Although primary neurons remain the gold standard for synaptic studies, they are constrained by limited availability, variability, and ethical concerns. Moreover, primary rodent neurons may not fully recapitulate all features of human neuronal circuits due to inherent species differences in synaptic organization, dendritic complexity, and electrophysiological properties. These limitations further underscore the need for standardized, accessible human-derived cell models such as SH-SY5Y.

Synapse formation is a key determinant of neuronal maturation and function. It is a prerequisite for cellular models intended to investigate synaptic structure and plasticity [12]. Synaptic model systems are vital to understanding signal transmission, circuit formation, and mechanisms underlying neurological disorders [13]. SH-SY5Y cells, derived from human neuroblastoma, have been extensively used to model neuronal differentiation, function, and disease [14, 15]. They are known to express synaptic proteins, exhibit neurite outgrowth, and evoke electrical activity or electrophysiological responses [16–19]. However, whether these features translate into the formation of mature synaptic structures remains unclear.

High-resolution techniques, such as transmission electron microscopy (EM) and cryo-electron tomography (cryo-ET), offer direct visualization of synaptic ultrastructure and are essential for confirming synapse formation [20, 21]. Despite their potential, these methods remain underutilized in the characterization of SH-SY5Y-derived neuronal models.

This gap is particularly relevant considering the numerous differentiation protocols designed to enhance the neuronal properties of SH-SY5Y cells. Differentiation of SH-SY5Y cells typically requires specific cell culture medium supplementation. The most common supplement is all-trans retinoic acid (RA), a factor promoting early neuronal development through the activation of nuclear receptors [22–25]. Supplementation with additional agents, such as phorbol esters and brain-derived neurotrophic factor (BDNF), enhances maturation and dopaminergic phenotype [26, 27]. Other supplements, such as dibutyryl-cAMP, promote neurite extension, while cholesterol supports synaptic vesicle formation [28–30]. Recent protocols combining RA, BDNF, and B-27^TM^ in neurobasal medium have also been reported to enhance neuronal differentiation of SH-SY5Y cells [16]. However, to our knowledge, there exists no direct ultrastructural evidence of bona fide synapses in SH-SY5Y cells to date.

In this study, we investigated the structure and functionality of SH-SY5Y cells differentiated over 28 days, including confocal immunofluorescence and live-cell imaging, protein expression analysis by Western blotting, conventional EM, cryo-ET, and whole-cell patch-clamp electrophysiology. We report that while differentiation increased synaptic protein expression, no protocol, including RA, BDNF, cAMP, or cholesterol supplementation, led to mature synapse formation. Additional neurotrophic factors did not significantly improve synaptogenesis on an ultrastructural level. This highlights the limitations of existing protocols to differentiate SH-SY5Y cells into a functional synaptic model successfully and underscores the need to explore alternatives.

## Results

### SH-SY5Y differentiation pattern and synaptic proteins upon differentiation

**Fig. 1:**
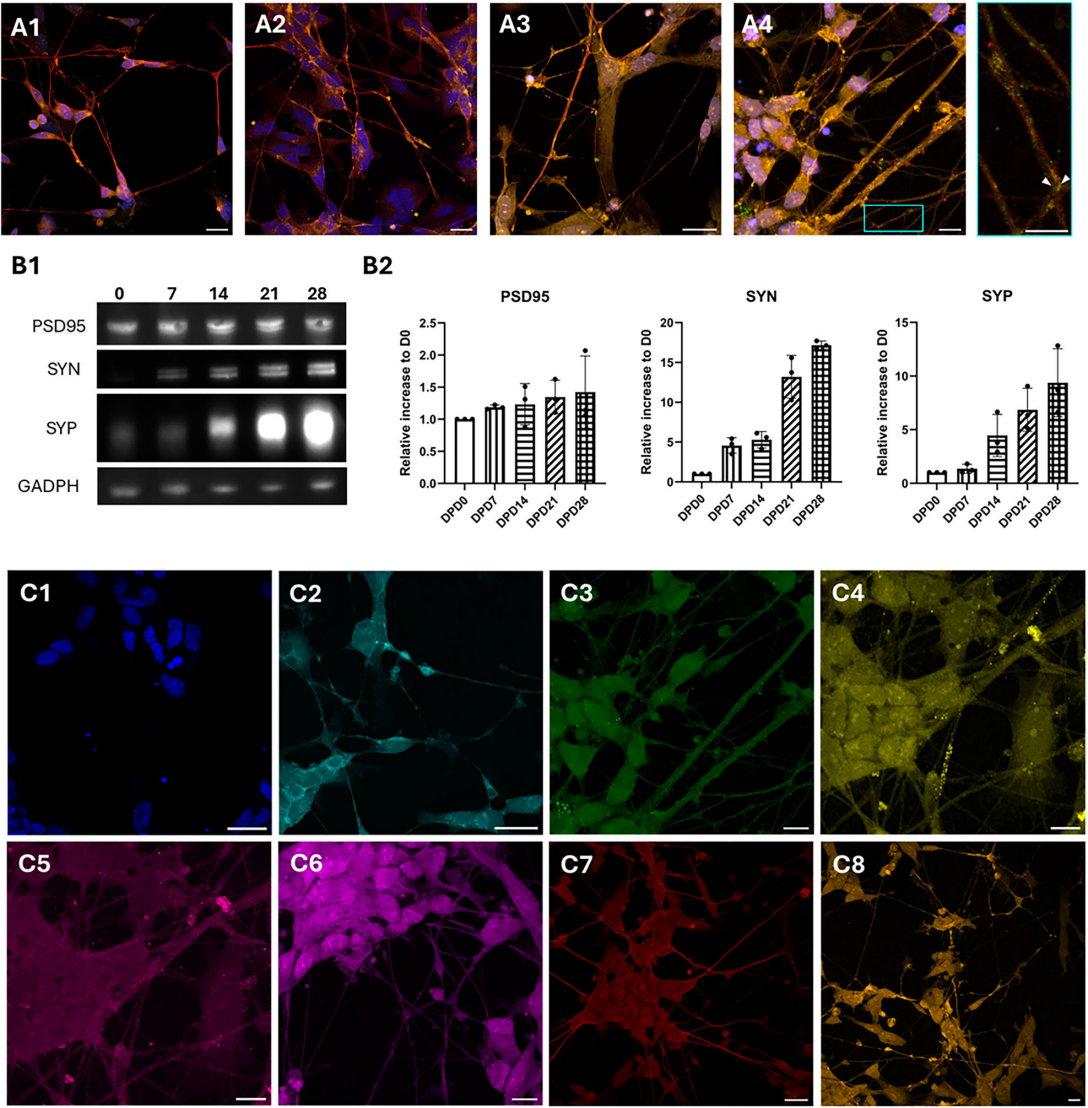
Relative increase in synaptic proteins. **A** Confocal images of SH-SY5Y cells over a 28-day differentiation period at timepoints DPD 7 (**A1**), DPD 14 (**A2**), DPD 21 (**A3**), and DPD 28 (**A4**) showing an increase of the presynaptic marker synaptophysin (orange); further, the postsynaptic marker PSD95 (green), the neurite marker β-tubulin III (red), and DAPI (blue) are shown. The zoomed-in square in cyan shows puncta-like structures (white arrows). Scale bars: 20 µm and 5 µm (zoom-in). **B1** Western blot of the synaptic proteins PSD95, synapsin I, and synaptophysin, as well as GAPDH (loading control) in undifferentiated cells (DPD 0) and over the course of differentiation (DPD 7-28). **B2** Semi-quantitative analysis of the relative protein expression of PSD95, synapsin I (SYN), and synaptophysin (SYP) normalized by GADPH and expression at DPD 0. Three individual biological replicates were used (*n* = 3). **C** Immunostaining of differentiated SH-SY5Y cells at DPD 28. **C1** DAPI, **C2** Synapsin I, **C3** PSD95, **C4** vGluT1, **C5** GluA2, **C6** Rab3a, **C7** β-tubulin III, **C8** Synaptophysin. Scale bars: 20 µm.

The formation of synaptic connections is an essential prerequisite for neurons to reach functional maturation [31]. Thus, we analyzed synaptic protein expression over time after initiating the differentiation process. SH-SY5Y cells were cultured and differentiated following established protocols, using differentiation media supplemented with cAMP, RA and BDNF for up to 28 days post-differentiation (DPD) [1]. The cells were fixed and fluorescently labeled with synaptophysin, PSD95, β-tubulin III, and DAPI and further processed for data acquisition at DPD 0, 7, 14, 21, and 28. The cells showed progressive neurite outgrowth over time (Fig. 1A1–4, S1–5 and S8), consistent with previous findings [32]. β-tubulin III, a neurite marker associated with mature neurons, displayed a homogenous distribution throughout all neurites (Fig. 1A1–4, Fig. S1–4, and S8) [33–35]. Synaptophysin, an integral membrane protein of synaptic vesicles (SVs) involved in vesicle trafficking and endocytosis, is widely used as a marker for presynaptic terminals [36]. It is endogenously expressed in SH-SY5Y cells and is observed to increase over time, as shown by both immunofluorescence microscopy (Fig. 1A1–4 and Fig. S5) and western blot analysis (Fig. 1B1). Quantitative western blot analysis (normalized to undifferentiated SH-SY5Y cells at day 0) revealed a 9.4 ± 2.5-fold increase in synaptophysin expression by DPD 28 (Fig. 1B2) [37]. Synapsin I, one of the most abundant presynaptic proteins in the brain, was detected at high levels, with a quantitative increase of up to 17.3 ± 0.5-fold relative to expression at DPD 0 (Fig. 1B2) [38]. In contrast, PSD95, a postsynaptic marker localized to the postsynaptic density and known to regulate excitatory synapse maturation, was observed in comparatively low levels (Fig. 1A1–4 and B1) [39]. Quantitative analysis revealed a modest 1.4 ± 0.5-fold increase over 28 days (Fig. 1B2).

A characteristic feature of mature neurons is the clustering of synaptic proteins along neurites and at sites of synaptic contacts (Fig. S7). When fluorescently labeling these clusters, they appear as discrete puncta. Labeling of both pre- and postsynaptic proteins typically shows puncta that are closely apposed or overlapping [40]. Synaptophysin displayed a mild punctate pattern, while PSD95 formed fewer visible puncta (Fig. 1A1–4; merged in S2–4 and Fig. 1C3–C8). However, little to no spatial proximity between pre- and postsynaptic puncta was observed (Fig. 1A4, zoom-in). PSD95 puncta were sparse at both DPD 21 and 28 (Fig. 1A3, A4, unmerged in Fig. S3 and Fig. S4), and their lack of overlap with synaptophysin puncta suggests a low degree of synapse formation, particularly when compared to mature neurons, as observed for example, in hippocampal cultures or in our primary cortical neurons and iPSC-derived neurons (Fig. S7) [41, 42].

Beyond synaptophysin, PSD95, and β-tubulin III, we examined the localization of additional key synaptic proteins to gain a more comprehensive overview of SH-SY5Y cell neuron-like characteristics. These include the presynaptic markers synapsin I, vGLuT1, and Rab3a, as well as the post-synaptic GluA2. Synapsin I exhibited a homogenous distribution throughout the cytoplasm with slightly elevated signal intensities at varicosities along neurites (Fig. 1C2). However, no apparent clustering into presynaptic puncta was observed. vGLuT1, a presynaptic vesicular transporter, showed localized puncta along neurites (Fig. 1C4) [43–45]. Rab3a, a small GTPase localized at presynaptic terminals, showed a diffuse distribution throughout the cytoplasm (Fig. 1C6) [46]. Similar to PSD95, GluA2, a subunit of AMPA receptors, primarily localized at postsynaptic sites, did not display punctae (Fig. 1C5) [47].

### SH-SY5Y cells differentiated by RA and BDNF do not form bona fide synapses

**Fig. 2:**
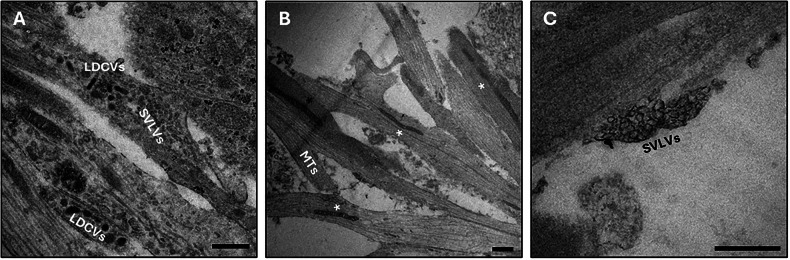
SH-SY5Y cell neuronal features in conventional electron microscopy. **A** Neurite extensions containing large dense-core vesicles (LDCVs). **B** Long tubulular mitochondria (*) in microtubule (MT) containing axon bundles. **C** Peripheral vesicle clusters with vesicles of similar size to SVs located along neurites. Scale bars: 200 nm.

**Fig. 3:**
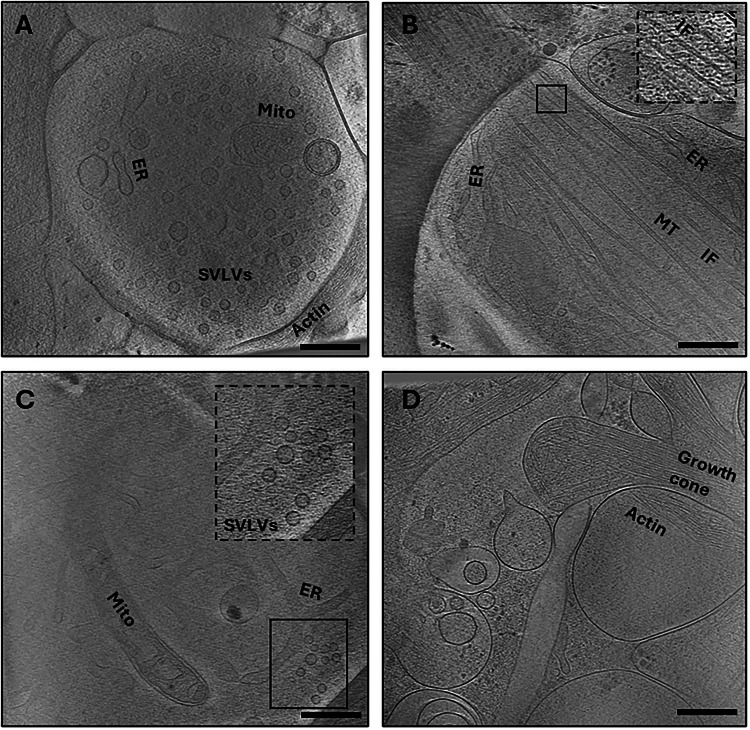
Cryo-electron tomogram of neuronal structures in SH-SY5Y cells. **A** Pre-synaptic-like bouton containing SVLVs, mitochondria, and endoplasmic reticulum (ER). **B** Axonal neurite with microtubules, tubular ER (likely smooth ER), and intermediate filaments (zoom-in). **C** Small cluster of SVLVs (zoom-in), mitochondria, and ER. **D** Growth cone with actin-filled filopodia of a newly forming neurite. Abbreviations: Mito: Mitochondria; ER: endoplasmic reticulum, SVLV: synapse-like synaptic vesicle, IF: Intermediate Filament; MT: Microtubule. Scale bars: 100 nm.

To investigate the ultrastructural features of differentiated SH-SY5Y cells, we analyzed neurites and their terminal specializations using both conventional EM and cryo-ET. Cells were either seeded in 6-well plates for conventional EM or cultured on EM grids for cryo-ET. We focused on neurites and their swellings, as well as putative axonal boutons, as potential sites of synaptic specialization (Figs. 2 and 3). We analyzed over 1000 conventional EM micrographs, more than 500 cryo-EM images, and over 500 cryo-ET reconstructions. These data revealed extensive neurite outgrowth and frequent cellular connections (Fig. 2C). SH-SY5Y axons typically exhibited a uniform diameter with densely packed microtubules and limited branching compared to dendrites (Fig. 2B). Interspersed among the microtubules were 10 nm wide intermediate filaments (Fig. 3B). Long tubular mitochondria, ranging from hundreds of nanometers to several micrometers and aligned with microtubules, were abundant within axonal neurites (Fig. 2B), a morphology characteristic of neuronal axons to support energy transport over extended distances [48]. Actin-rich membrane protrusions, lacking microtubules, were identified at neurite tips and branching points, indicating the presence of filopodia and growth cones (Fig. 3D). These cytoskeletal and organellar features, including the tubular mitochondria and endoplasmic reticulum (ER; involved in axonal calcium handling) are consistent with early axon organization in primary hippocampal neurons, though such organelles are not exclusive to neurons [49–51]. Dendrites, identified by their larger diameter, the presence of spines, polyribosome clusters, and a more loosely organized cytoskeleton, were less frequently observed [49].

During differentiation, we observed fine cellular protrusions between SH-SY5Y cells, particularly at earlier points (DPD 14), which resemble tunneling nanotubes (TNTs) (Fig. S14) [52]. These structures were less prominent at later stages (DPD 28), when neurite-like processes dominated morphology. Although these protrusions superficially resemble structures implicated in intercellular interactions, such as thin membrane bridges, we did not assess cytoskeletal composition, membrane continuity, or intercellular transport, and therefore refrain from making further interpretations regarding their identity or function [53, 54]. Nevertheless, their presence in early-stage cultures may indicate dynamic morphological features of SH-SY5Y cells that warrant further investigation.

In primary neurons and synaptosomes, mature synapses are structurally characterized by a presynaptic bouton containing clusters of small, clear SVs, often arranged in distinct pools and interconnected by filamentous connectors. These boutons feature a defined active zone aligned with the synaptic cleft and a postsynaptic compartment, typically a dendritic spine [20, 55]. In differentiated SH-SY5Y cells, presynaptic-like structures were observed at axonal boutons containing SVLVs. These vesicles had a mean diameter of approximately 35 nm, which is slightly smaller than typical SVs ( ~ 45 nm) (Figs. 2C, 3A, C). The observed SVLVs were regularly organized in clusters but appeared to be devoid of any connectors (Fig. 3C, zoom-in), which are typically found in SV pools. Additionally, the presynaptic-like bouton lacked the characteristic active zone and a postsynaptic counterpart (Fig. 3A, C). Similarly, varicosities along axons containing SVLVs along with mitochondria and tubular ER are abundantly present, organelles that, while ubiquitous, show morphologies relevant to neuronal axons (e.g., elongated mitochondria supporting energy demands in extended processes). However, at these varicosities, no typical SV architecture, i.e., organized pools and connectors were observed (Fig. 2C). The SVLVs were of pleomorphic shape and size. We further observed multivesicular bodies (MVBs), which may be easily mistaken for presynaptic terminals due to their small vesicles enclosed in a large membrane, but are distinguishable by the protein decoration on the vesicles (Fig. S11). Large dense core vesicles (LDCV), presenting as 100 to 300 nm dark vesicles, were also seen in neurites as well as in close proximity to SVLVs (Fig. 2A). As cholesterol has been implicated in aiding SV formation, we further investigated SH-SY5Y cells differentiated using this approach through high-throughput screening of EM sections (Fig. S9).

### Adding additional neurotrophic factors did not further synapse maturation

We attempted to trigger a more mature neuronal state by supplementing the culture with additional neurotrophic factors (NTFs), namely glial-derived neurotrophic factor (GDNF), ciliary neurotrophic factor (CNTF), and insulin-like growth factor (IGF-1), which were added after DPD 14. This combination is inspired by protocols that promote functional maturation in neuronal differentiation models, including iPSC-derived neurons (Fig. S7) [56]. Neurotrophic support, particularly involving GDNF, CNTF, and IGF-1, is well-established to promote neuronal survival, differentiation, and maturation in various models, including neuroblastoma-derived lines [37, 57, 58].

**Fig. 4:**
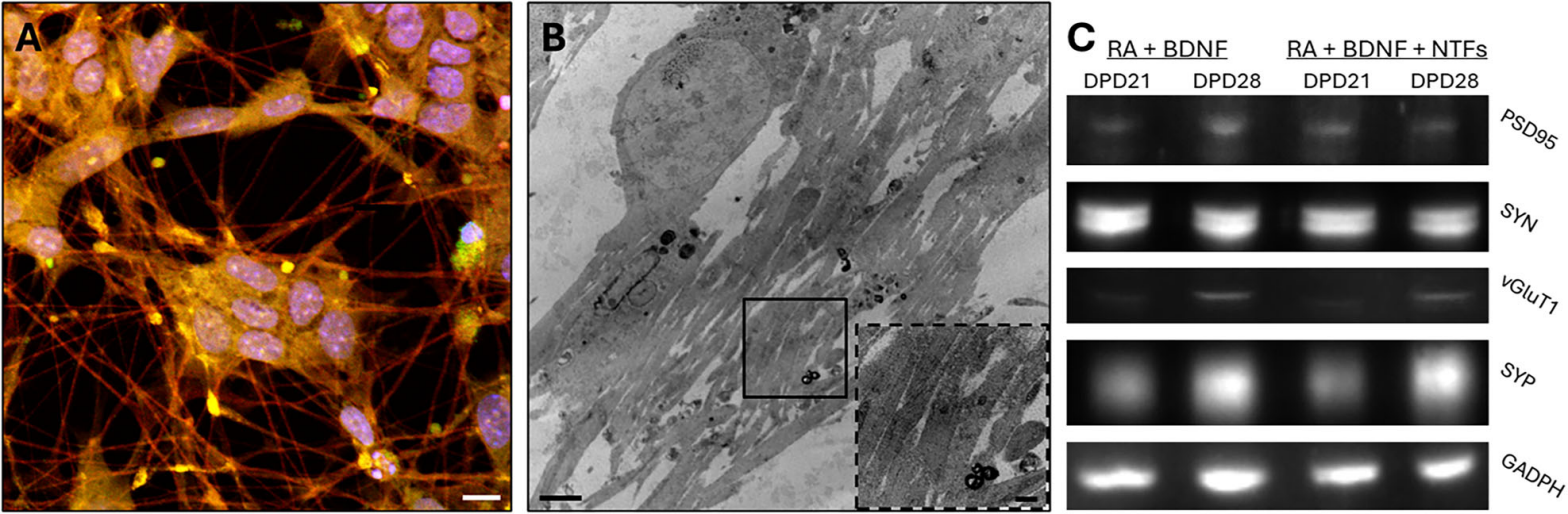
Analysis of differentiation by RA + BDNF with additional NTFs. **A** Confocal image of NTF-differentiated SH-SY5Y cells stained with synaptophysin (orange), PSD95 (green), β-tubulin III (red), and DAPI (blue). Immunofluorescence stain shows no clear synaptic puncta. Scale bar: 10 µm. **B** Conventional EM micrograph of neurites and zoom-in of axonal endings. Scale bars: 500 nm and 200 nm. **C** Western blot analysis of synaptic protein expression levels in RA + BDNF-differentiated compared to NTF-differentiated SH-SY5Y cells at DPD 21 and 28 of synaptophysin (SYP), synapsin-I (SYN), PSD95, vGluT1, and GADPH (loading control) showed no significant difference in protein expression level between the differentiation methods.

These SH-SY5Y cultures were subjected to protein profile analyses using fluorescent staining and western blotting, as well as ultrastructural investigations and functional assays. Comparative Western blot analysis revealed a modest increase in vGluT1 and synaptophysin levels from DPD21 to DPD28 in cells differentiated with RA + BDNF alone as well as those supplemented with additional NTFs, with no significant differences between the two conditions (Fig. 4C). Morphological analysis by immunofluorescence (Fig. 4A, unmerged in Fig. S13) revealed that SH-SY5Y cells treated with additional NTFs tended to exhibit more separated neurites in contrast to the prominent axon bundling observed with BDNF and RA alone (Fig. 1A1–4, Fig. S8). EM analysis revealed that, despite the presence of additional NTFs, presynaptic-like structures lacked a postsynaptic counterpart, including SVs, an AZ, and vesicle connectors (Fig. 4B).

### Standard differentiation methods cannot achieve synaptic activity

**Fig. 5:**
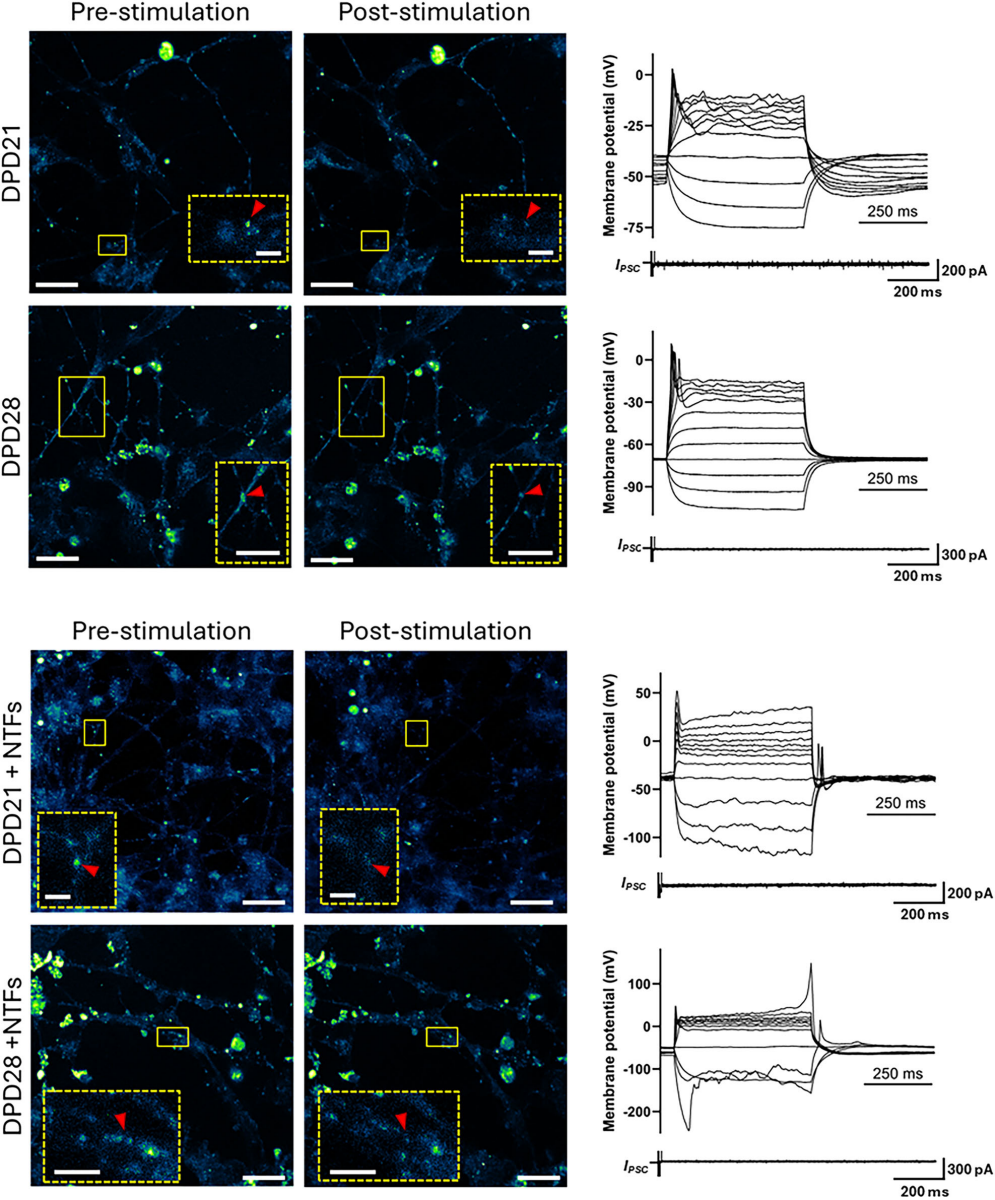
Functional live-stain assay with AM4-64 dye and electrophysiological traces. Fluorescence images of SH-SY5Y cells differentiated with either BDNF + RA at DPD 21 and 28 or with additional NTFs at DPD 21 and 28, stained with AM4-64 dye. Z-projected images of ROI with small zoom-ins on puncta pre-stimulation (left side) and z-projected images with zoom-ins on puncta acquired post-stimulation with KCl after 120 s (right side). Zoom-ins show the movement of labeled vesicles (red arrow). Scale bars: 10 µm and 2 µm (zoom-ins). Respective current-clamp mode recordings (right side on top) to the corresponding immunofluorescence images demonstrated singular action potentials in response to stepwise depolarizing currents. x-axis shows time (ms); y-axis shows membrane potential (mV). Voltage-clamp recordings (right side on the bottom) showed no spontaneous post-synaptic potentials (sPSPs), suggesting no signals received from neighboring cells. x-axis shows time (ms); y-axis shows spontaneous currents (pA).

To evaluate the functional maturation of SH-SY5Y cells, we performed whole-cell patch clamp recordings in both current clamp and voltage clamp configurations across four experimental conditions, DPD 21 and DPD 28, with and without neurotrophic factor supplementation. Current-clamp recordings demonstrated single action potentials in response to stepwise depolarizing current injections (from -60 to +160 pA in 20 pA steps) (Fig. 5). Voltage-clamp recordings, holding the cell at -60 mV, showed no spontaneous postsynaptic potentials, indicating a lack of detectable spontaneous synaptic activity (Fig. 5). Notably, even prolonged differentiation and trophic support using NTFs failed to induce voltage-gated conductances or membrane excitability.

To further assess stimulus-evoked exocytosis, we performed live-cell imaging using the fluorescent dye AM4-64, which binds reversibly to the plasma membranes and is internalized by endocytic events. Subsequent quenching of non-internalized dye enables visualization of endocytosed vesicles. In certain neuronal synapses, recently endocytosed vesicles have a higher probability of undergoing evoked exocytosis [59]. SH-SY5Y cells at DPD 21 and DPD 28 were imaged after dye loading and quenching at 2-second intervals during high-potassium stimulation. While some fluorescent puncta were observed in neurites at DPD 21 and increased in number at DPD 28, their apparent disappearance following stimulation was not due to vesicle exocytosis (Fig. 5). Instead, time-lapse imaging revealed that vesicles remained fluorescent but moved along neurites and frequently shifted in and out of the imaging plane, thus mimicking fluorescence loss (Fig. S12 and Fig. S19–22). The observed endocytosed vesicles did therefore not show the capacity for swift exocytosis upon repeat stimulation, as is readily observed in neuronal SVs.

## Discussion

In this study, we evaluated the neuronal properties of SH-SY5Y cells differentiated using established protocols, with or without supplementation with additional neurotrophic factors over a 28-day culture period, to assess their suitability as an in-vitro neuronal model.

Differentiated SH-SY5Y cells exhibit several neuronal features, including rapid neurite outgrowth, expression of β-tubulin III, and ultrastructural distinctions between axon- and dendrite-like structures. Organelles relevant to neuro- and synaptogenesis, such as mitochondria and ER, are present, and cytoskeletal elements (intermediate filaments, actin-rich growth cones) align with early neuronal organization. However, the cells do not recapitulate the structural and functional aspects characteristic of mature neurons in terms of synapse formation.

The expression of presynaptic and postsynaptic markers under standard differentiation protocols (RA, BDNF, cAMP, and B27) is consistent with partial neuronal maturation, yet the diffuse distribution of most synaptic proteins and lack of overlapping pre- and postsynaptic puncta reflect an immature phenotype. In immature neurons, synaptic proteins are often synthesized but not fully trafficked to synaptic sites, resulting in the absence of the punctate patterning typical of mature neurons [60].

The organization of cytoskeletal elements provides insight into the neuronal maturity of SH-SY5Y cells. Ultrastructural analysis confirmed the presence of intermediate filaments, which are essential for maintaining axonal integrity and function [61]. SH-SY5Y cells also exhibited actin-rich growth cones and distinct axon- and dendritic-like structures. In mature neurons, such cytoskeletal specializations are essential for synaptic function and neurite stability [62]. Closer examination of the cytoskeleton in Cryo-EM revealed thin extensions containing straight actin bundles, consistent with tunneling nanotubes (TNTs), which transport cellular material between cells (Fig. S14) [52]. These extensions were more abundant at early differentiation time points and were gradually replaced by axon-like neurites composed of microtubules upon further differentiation.

Despite extensive neurite outgrowth, SH-SY5Y cells failed to form functional synaptic connections. Next to LDCVs, SVLVs were present at neurite terminals. However, they did not assemble into organized pools typically associated with mature presynaptic boutons [20, 55, 63–65]. LDCVs were also observed. Taken together with the diffuse non-punctate distribution of synaptic proteins, these findings suggest that SH-SY5Y cells are unable to initiate synaptogenesis.

To further promote synaptogenesis, we differentiated SH-SY5Y cells in the presence of additional neurotrophic growth factors (GDNF, CNTF, IGF1), which have been reported to support neuronal maturation, especially in iPSC-derived neurons [11, 66]. However, biochemical and structural analyses revealed no significant enhancement in synaptic protein expression levels or patterns, or in synaptic ultrastructure. Electrophysiological recordings showed that SH-SY5Y cells, despite additional NTF supplementation, exhibited only single action potentials, reflecting limited excitability likely due to insufficient ion channel density or overall neuronal immaturity [67–69]. Such isolated action potentials are typical of immature neurons, in which ion channel kinetics and synaptic inputs are not yet fully developed [70].

To investigate potential endocytic and exocytic activity, we used dye AM4-64 [71]. Fluorescent puncta, corresponding to recently endocytosed vesicles, were observed in neurites, but rather than undergoing exocytosis upon repeat stimulation, they exhibited directional movements along neurites [72, 73]. This behavior reflects intracellular transport dynamics rather than synaptic vesicle recycling [74]. As AM4-64 labels all membrane-bound compartments resulting from endocytosis, including early and recycling endosomes, lysosomes, and transport vesicles, the observed motility could be attributed to the trafficking of these organelles [75, 76].

One possible explanation for the observed failure of SH-SY5Y cells to undergo synaptogenesis is a deficiency in target recognition mediated by cell adhesion molecules such as cadherins and integrins, which are known to be essential for establishing and stabilizing initial pre- and postsynaptic contacts in primary neuronal models [77]. However, this remains speculative and was not directly tested in the present study; future experiments, such as cadherin/integrin expression analysis or functional perturbation, could address this possibility.

In conclusion, our study demonstrates that SH-SY5Y cells differentiated with RA and BDNF fail to form synapses. Although these cells display several neuronal characteristics, their lack of functional synaptic connections and their immature electrophysiological properties limit their utility as a model for synaptic studies.

## Methods

### Cell culture

SH-SY5Y cells were maintained in Eagle’s minimum essential medium (EMEM, Gibco, Thermo Fisher Scientific), supplemented with 10% heat-inactivated fetal bovine serum (hiFBS, Gibco) and 1% penicillin-streptomycin (P/S, Gibco) in T-75 flasks, incubated at 37 °C 5% CO_2_. The cells were passaged at a 1:10 ratio every week using 3 mL 0.25% Trypsin-EDTA (1X) (Gibco, Thermo Fisher Scientific). Glass coverslips (BRAND, Sigma-Aldrich) and holey carbon-coated gold EM grids (300 mesh with Quantifoil R 2/1 or 200 mesh with lacey carbon film, EMS) were placed into dishes and were pre-coated with diluted (1:20 with ddH2o) 0.01% Poly-L-Lysin (PLL, Sigma-Aldrich) [14]. Cells were seeded at a density of 100,000 cells per well of a 6-well plate in EMEM supplemented with 10% hiFBS, 2 mM Glutamine, and 1% P/S. They were further differentiated using the media compositions adapted from Shipley et al. (2016). At day 3, the medium was exchanged to differentiation medium #1 (EMEM, 2.5% hiFBS, 2 mM Glutamine, 1% P/S 10 µM RA (Sigma-Aldrich)). At day 5, differentiation medium #2 (EMEM, 1% hiFBBS, 1% P/S, 2 mM Glutamine, and 10 µM RA) was introduced. At day 7, differentiation medium #3 Neurobasal (Gibco, Thermo Fisher Scientific), 1X B-27 (Gibco, Thermo Fisher Scientific, 20 mM KCl, 1% P/S, and 2 mM Glutamax (Gibco, Thermo Fisher Scientific)) was used and maintained until day 14. On day 14, SH-SY5Y cells were further differentiated by adding 50 ng/mL BDNF (STEMCELL) or 50 ng/mL BDNF, 10 ng/mL GDNF (STEMCELL), 10 ng/mL CNTF (STEMCELL), and 10 ng/mL IGF1 (STEMCELL) to medium #3. The medium was exchanged every three days until day 28.

### Immunofluorescence

Cells grown on coverslips were removed from the dish and fixed with 3% paraformaldehyde and 0.5% glutaraldehyde (Agar Scientific, UK) for one hour at room temperature (RT), followed by washing in PBS three times. The cells were permeabilized with 0.3% Triton X-100 (Sigma-Aldrich) for 15 min and washed again with PBS. 3% SureBlock (LubioScience) in PBS was used for unspecific blocking for one hour at RT. The cells were labeled with primary antibodies overnight and subsequently secondary antibodies (see Supplemental Table) in 3% blocking agent for one hour at RT, washing in-between 3 × 5 min with PBS. The coverslips were dried overnight at 4 °C. The coverslips were mounted on glass slides using Antifade mounting medium (ProLong Glas, Thermo Fisher Scientific) and were stored at 4 °C until imaged. The specificity of the secondary antibodies used in this study was ensured by secondary-only stains (Fig. S6)

### Immunoblotting

For immunoblots, SH-SY5Y cells were cultured as described above on a 60 mm dish at a seeding density of 250,000 cells per dish. The cells were washed twice with PBS before being added to 400 µL of RIPA buffer containing 1:100 EDTA-free proteinase inhibitor (Sigma-Aldrich). They were lysed for 30 min on ice on a shaker. They were then detached with a cell scraper and transferred into 1.5 mL tubes. The lysate was then sonicated 3 × 10 s at 50% power using the SONOPLUS HD2070 homogenizer (Bandelin) and incubated on ice for a further 10 min. The fully lysed cells were centrifuged at 14.5 x *g* for 20 min, and the supernatant containing the proteins of interest were collected. The protein content was determined using a bicinchoninic acid assay (Sigma-Aldrich). The samples were prepared in Laemmli buffer, boiled for 5 min at 95°C, snap-frozen, and stored at –80 °C. The samples were equilibrated to 5 µg/well and loaded onto a 10% bisacrylamide gel. The gel was run for 30 min at 20 mA, followed by 90 min at 40 mA. The gel was then transferred onto a nitrocellulose membrane (Merck) for 70 min at a constant 100 V. The membrane was washed with PBS with 1% Tween (PBST) for 10 min and then incubated with Intercept (PBS) Blocking Buffer (LI-COR Biosciences) in PBST for one hour. The primary antibodies (see Supplemental Table 1) in the blocking buffer were added overnight, the membranes were then washed 3x with PBST and further incubated with HRP-conjugated secondary antibodies (See Supplementary Table 1) for one hour. The washed membranes were incubated with ECL Reagent (Thermo Fisher Scientific) and detected using the Fusion Fx system (Vilber Lourmat). Western blot images were quantified using GelAnalyzer and compiled in Excel (Microsoft Corporation).

### Vesicle recycling assay

The vesicle recycling assay was adapted from Gaffield & Betz (2007) and Iwabuchi et al. (2014) using the FM dye AM4-64 (Biotium, VWR International) [78, 79]. To enable gentle medium exchange during imaging, a custom-made perfusion system using 20-gauge needles was adapted to a µ-dish (ibidi). The perfusion system was installed in the LSM 880 confocal microscope (Zeiss). Cells were washed in Tyrode’s solution (124 mM NaCl, 5 mM KCl, 2 mM CaCl_2_, 1 mM MgCl_2_, 30 mM glucose, 25 mM HEPES; 310 mOsm/l and pH 7.4) and then incubated for 2 min in Tyrode’s solution containing 10 µM AM4-64 dye. The cells were stimulated for 2 min with a depolarizing high-potassium solution (Tyrode’s containing 70 mM KCl, 59 mM NaCl, and 10 µM AM4-64). This was followed by a 10 min incubation in standard Tyrode’s solution containing 10 µM AM4-64 to allow slow endocytosis staining [31]. Non-internalized dye was quenched for 5 min with 0.5 mM SCAS in low-calcium and high-magnesium Tyrode’s solution (0.2 mM CaCl_2_ and 5 mM MgCl_2_), which halts spontaneous re-exocytosis of the endocytosed vesicles. A z-stack was acquired at the region of interest. Then the cells were stimulated with high-potassium Tyrode’s solution, and a micrograph was acquired every 2 s for 3 min using a large pinhole (5.42 AU), thus broadening fluorescence detection in the z-plane (0.99 μm focus depth) using the laser at 488 nm and 1.8% strength. This allowed us to minimize laser intensity and reduce photobleaching. A z-stack of the region of interest was acquired after stimulation.

### Whole-cell patch-clamp electrophysiology

SH-SY5Y cells were cultured on 12 mm round glass coverslips (Fisher Scientific) and were transferred to a recovery chamber filled with oxygenated and carbonated artificial cerebrospinal fluid (aCSF) containing 125 mM NaCl, 2.5 mM KCl, 25 mM NaHCO_3_, 1.25 mM NaH_2_PO_4_, 1 mM MgCl_2_, 2 mM CaCl_2_, and 25 mM glucose, kept at RT. Whole-cell patch-clamp recordings were performed using heat-pulled borosilicate glass pipettes with a tip resistance of 4–9 MΩ. The cells were visualized through an infrared charge-coupled detector camera mounted on a Leica DM LFSA microscope (Thorlabs). Cells were subjected to 500 ms square current injections ranging from -60 to 300 pA in 20 pA increments. Custom-made Igor Pro procedures (WaveMetrics) were used for all data acquisition and analyses [80].

### Electron microscopy of resin-embedded samples

Cells grown on 6-well plates were fixed in 0.15 M HEPES containing 2.5% glutaraldehyde (670 mOsm, pH 7.35) (Fluka) at 4°C for a minimum of 24 h. They were then washed with 0.15 M HEPES 3 × 5 min, post-fixed with 1% OsO4 (EMS) in 0.1 M Na-cacodylate buffer (Merck) at 4 °C for one hour. Then, the cells were washed in 0.1 M Na-cacodylate buffer 3 × 5 min and dehydrated through a graded ethanol series (70, 80, 96, and 100% ethanol), each for 15 min at RT. Subsequently, the samples were infiltrated with a 1:1 mixture of ethanol and Epon 812 (Fluka) overnight at RT. They were then embedded in pure Epon 812 and left to harden at 60 °C for 5 days. The resin blocks were removed from the dishes and sectioned with a UC6 ultramicrotome (Leica Microsystems), starting with semi-thin sections of 1 µm thickness, which were stained with a 0.5% toluidine blue O solution (Merck). Ultrathin sections of 70 nm thickness were cut with the ultra-diamond knife 45° (DiATOME). The sections were mounted on uncoated 200 mesh copper grids (G2200C, Plano GmBH), stained with uranyLess (EMS) and 3% lead citrate (Leica) using the EM STAIN device (Leica Microsystems, Vienna, Austria). The sections were imaged using a Tecnai Spirit transmission electron microscope (Thermo Fisher Scientific) equipped with a Veleta CCD camera (EMSIS) at an accelerating voltage of 80 kV.

### Cryo-electron microscopy and tomography

SH-SY5Y cells were cultured on poly-L-lysine-coated holey carbon film EM grids: R 2/1 – 300 mesh (Quantifoil) or lacey carbon film—200 mesh (EMS). After transferring the grid to a custom-made plunge freezer, 4 µL of a solution containing 10 nm colloidal gold beads as fiducial markers was added. The grids were manually blotted using 12/N filter paper (Munktell) and vitrified by rapid plunging into liquid ethane, then stored in liquid nitrogen. Cryo-EM micrographs were acquired using the FEI Tecnai F20 (200 kV), with an FEI Falcon 2 direct detector. Tomograms were acquired on a Titan Krios G4 transmission electron microscope (Thermo Fisher Scientific) operating at 300 kV and ×26,000 magnification. Tilt series were recorded from ±60° with 2° or 3° increments using the tomo5 software. The total electron dose for each projection of the tilt series was adjusted to 1–1.55 e⁻/Å², and images were collected at a target defocus of −10 µm. Data acquisition was performed using a Falcon 4i direct electron detector in combination with a Selectris energy filter. The tomograms were reconstructed using IMOD [81].

### Statistics and reproducibility

No animal or human subjects were used in this study. All experiments were performed using the human neuroblastoma cell line SH-SY5Y. A minimum of ten independent cell culture preparations (biological replicates) were generated across different passages and differentiation batches to ensure reproducibility of morphological, ultrastructural, and molecular observations. All independent culture preparations that met predefined technical quality criteria (e.g., absence of contamination, normal morphology, and successful differentiation according to the described protocol) were included in the analysis. For immunofluorescence (IF), western blot (WB), live exocytosis assays, and electrophysiological recordings, at least three independent biological replicates (derived from separate culture preparations) were analyzed. Where applicable, multiple technical replicates were acquired per biological replicate (e.g., multiple fields of view per coverslip for IF; multiple cells per recording session for electrophysiology). Conventional electron microscopy and cryo-electron tomography (cryo-ET) analyses were performed across at least ten independent cultures to ensure consistent ultrastructural observations. Western blot analyses are presented with individual data points representing independent biological replicates. Due to the absence of mature synaptic structures in this model system, several analyses are presented descriptively rather than as quantitative statistical comparisons. Where quantification was performed, values are reported as mean ± SEM unless otherwise stated. No statistical methods were used to predetermine sample size. Experiments were not randomized and investigators were not blinded during data acquisition.

## Supplementary information

Supplemental Materials

Tomogram_of_Fig_3A

Tomogram_of_Fig_3B

Tomogram_of_Fig_3C

Tomogram_of_Fig_3D

Video_of_Fig5_Livestain_D21_RA_BDNF

Video_of_Fig5_Livestain_D28_RA_BDNF

Video_of_Fig5_Livestain_D21_RA_BDNF_NTFs

Video_of_Fig5_Livestain_D28_RA_BDNF_NTFs

Tomogram_of_Fig_S8B

Dataset2 (FL-WBs)

Row Excel data for Fig. B2

## Data Availability

All data generated or analyzed during this study are included in this published article and its supplementary information files. Raw confocal and electron microscopy images, uncropped western blots, and electrophysiological recordings are available from the corresponding authors upon reasonable request.
