# Supplementary material for: Differentiated SH-SY5Y cells exhibit neuronal features but lack synaptic maturity
Source: Cell Death Discov. 2026 Apr 14;12:302. doi: 10.1038/s41420-026-03094-y (PMC13369904; doi:10.1038/s41420-026-03094-y)

Full size WBs for Figure 1B1, 1B2, and 4

WBs of PSD95 for Fig 1B1 and 1B2

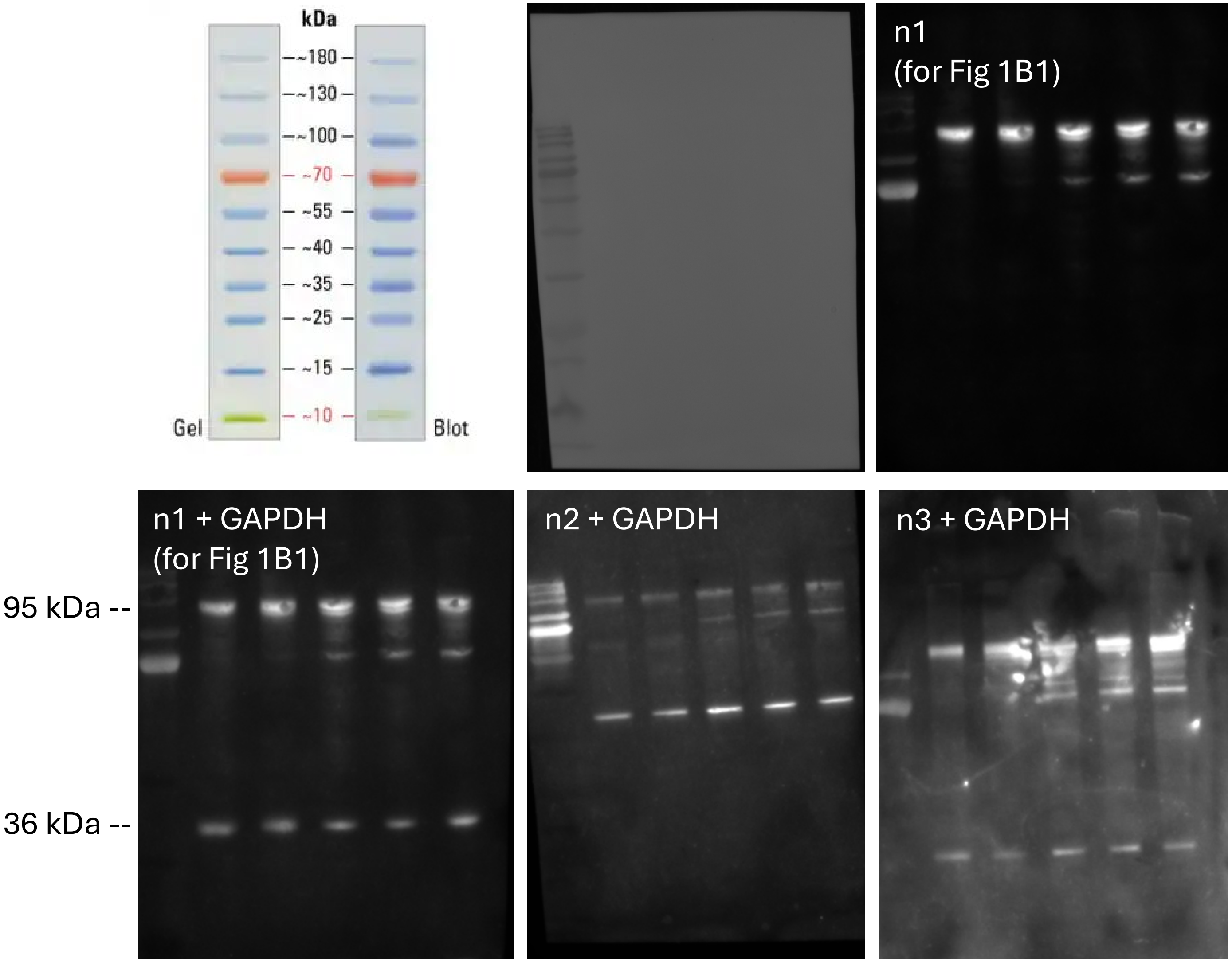

WBs of SYP for Fig 1B1 and 1B2

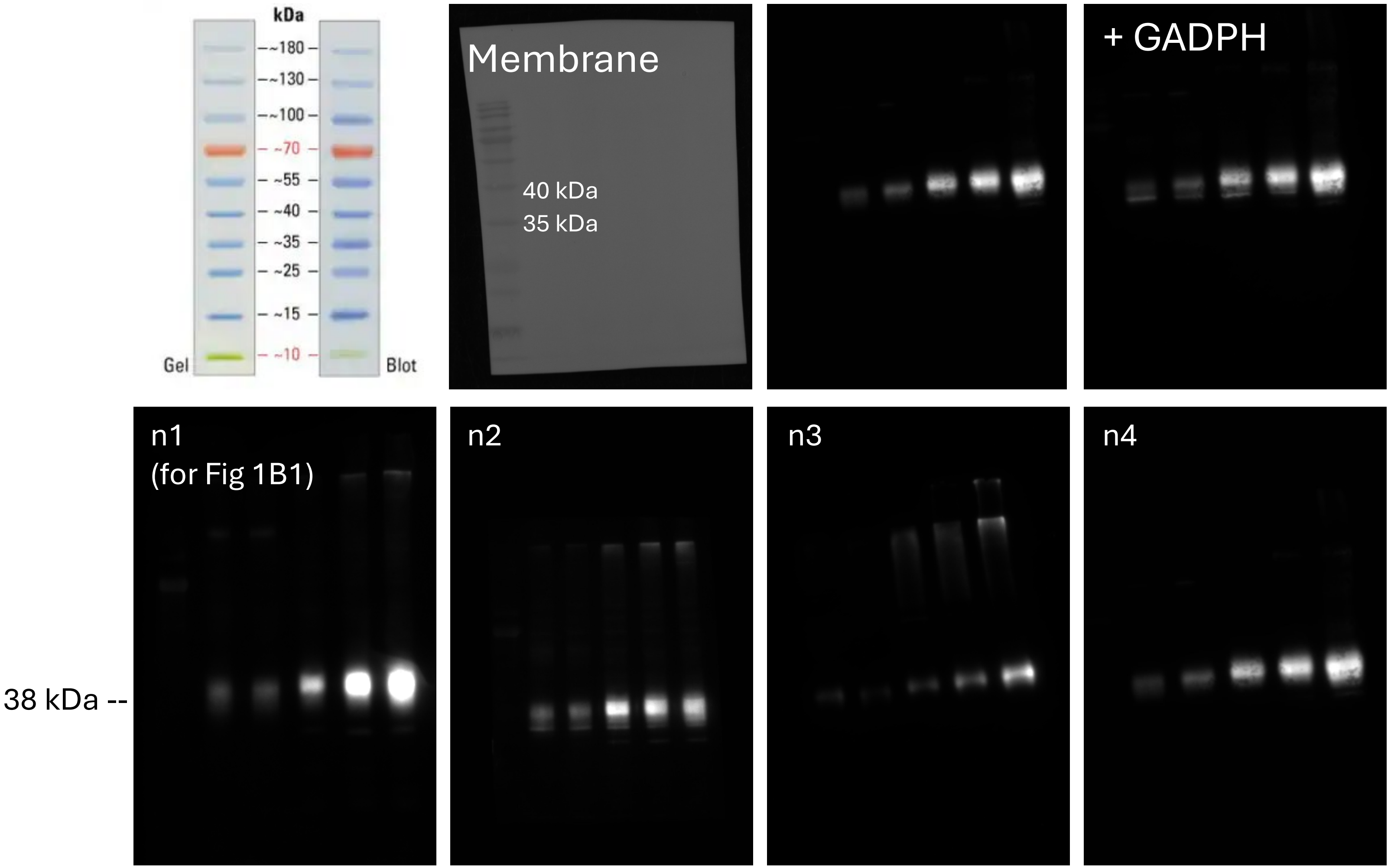

WBs of SYN for Fig 1B1 and 1B2

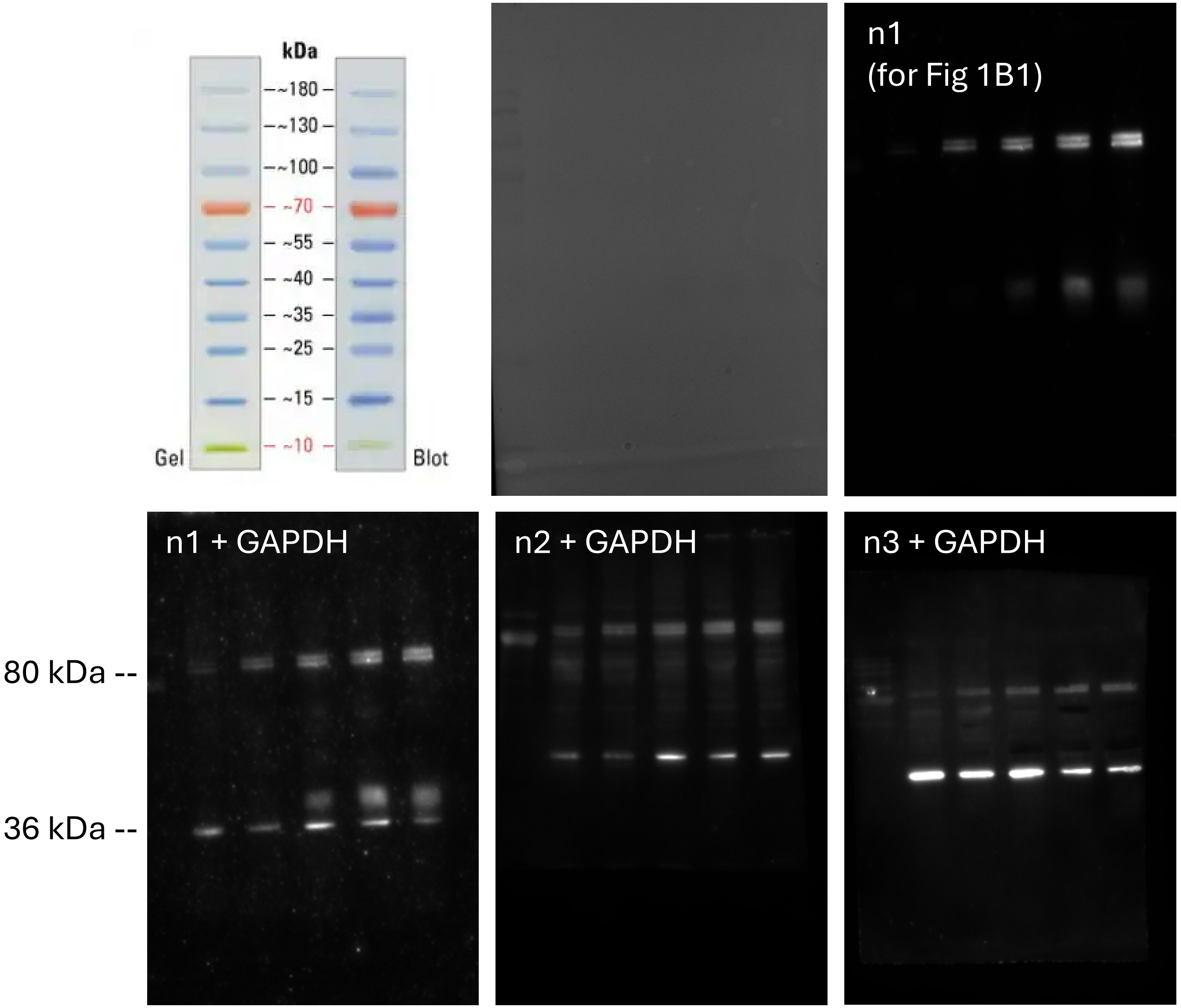

WBs of proteins for Fig 4

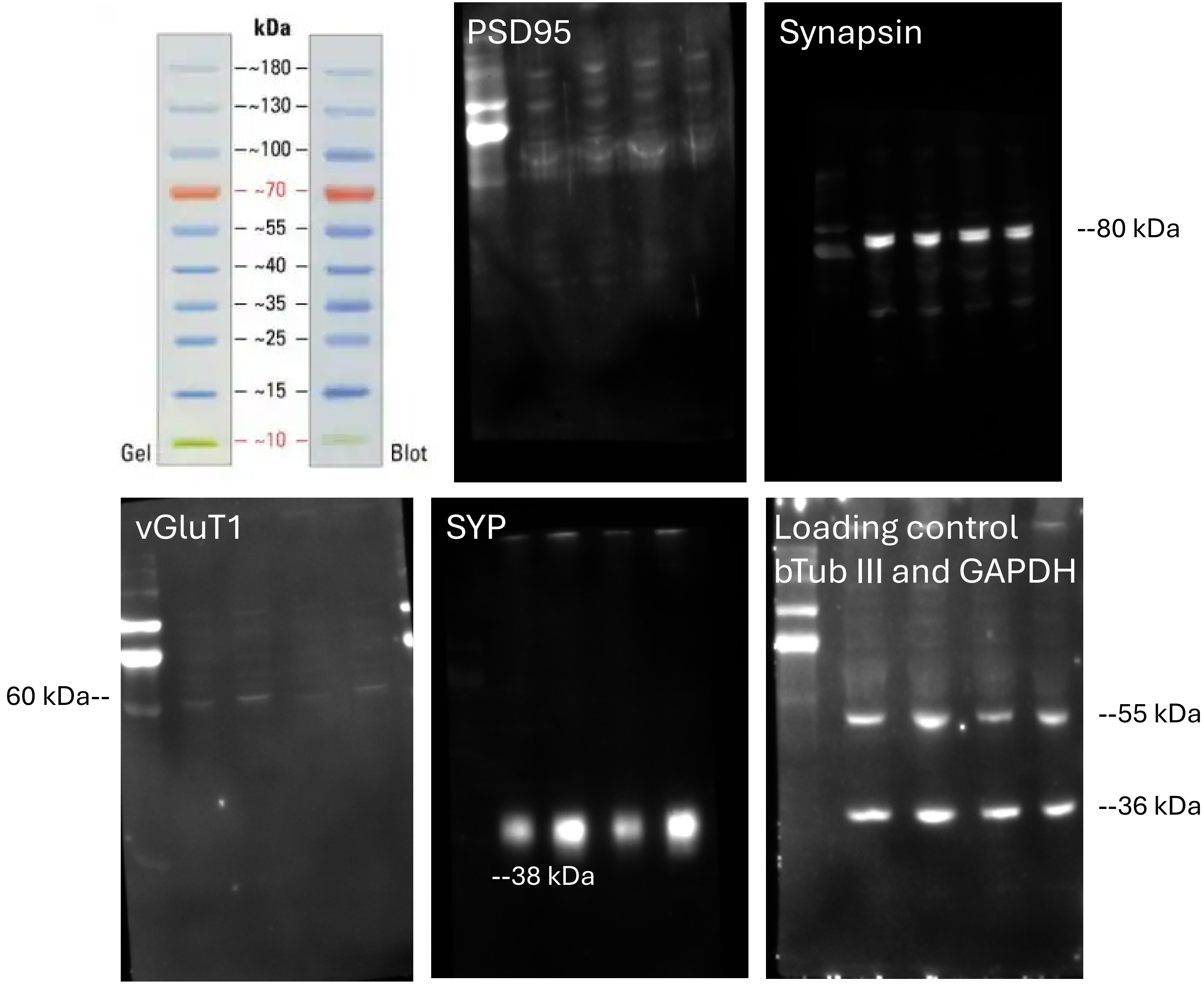

Supplement: Supplementary file 11 — Dataset2 (FL-WBs) [file 41420_2026_3094_MOESM11_ESM.pdf]
