# Supplementary material for: Differentiated SH-SY5Y cells exhibit neuronal features but lack synaptic maturity
Source: Cell Death Discov. 2026 Apr 14;12:302. doi: 10.1038/s41420-026-03094-y (PMC13369904; doi:10.1038/s41420-026-03094-y)
Supplement: Supplementary file 1 — Supplemental Materials [file 41420_2026_3094_MOESM1_ESM.docx]

# Supplemental Materials

Table 1: Primary and secondary antibodies and dyes used

| Type | Descriptor / Conjugate | Manufacturer | Product number | Host | Dilutions used for IF / WB |
| --- | --- | --- | --- | --- | --- |
| Primary Antibodies | Anti-Synaptophysin | Sigma-Aldrich | S5768 | Mouse monoclonal | 1:1000 / 1:5000 |
|  | Anti-Synapsin I | Sigma Aldrich | S193 | Rabbit polyclonal | 1:1000/ 1:5000 |
|  | Anti-Rab3a | Proteintech | 16865-1-AP | Rabbit polyclonal | 1:1000 / 1:5000 |
|  | Anti-vGluT1 | Synaptic Systems | 125511 | Mouse monoconal | 1:1000/ 1:5000 |
|  | Anti-PSD-95 | Abcam | ab238513 | Rabbit monoclonal | 1:1000 / 1:1000-1:5000 |
|  | Anti-PSD-95 | Invitrogen | MA5-45141 | Mouse monoclonal | 1:1000 / - |
|  | Anti-MAP2 | Invitrogen | PA1-10005 | Chicken polyclonal | 1:1000 / 1:5000 |
|  | Anti-GluA2 | Sigma Aldrich | ZRB1008 | Rabbit monoclonal | 1:1000/ 1:5000 |
|  | Anti-β-III-tubulin | Novus Biologicals | NB100-1612 | Chicken polyclonal | 1:1000 / - |
|  | Loading control: Anti-β-tubulin / HRP | Invitrogen | MA5-16308-HRP | Mouse monoclonal | - / 1:5000 |
|  | Loading control: Anti-GAPDH / HRP | Invitrogen | MA5-15738-HRP | Mouse monoclonal | - / 1:5000 |
| Secondary Antibodies | Anti-Rabbit / Alexa-488 | Invitrogen | A11004 | Goat polyclonal | 1:1000 / - |
|  | Anti-Goat / Alexa-488 | Invitrogen | A11055 | Donkey Polyconal | 1:1000 / - |
|  | Anti-Gunea Pig / Alexa-488 | Invitrogen | A11073 | Goat Polyclonal | 1:1000 / - |
|  | Anti-Rabbit / Alexa-568 | Invitrogen | A11011 | Goat polyclonal | 1:1000 / - |
|  | Anti-Mouse / Alexa-568 | Invitrogen | A11004 | Goat polyclonal | 1:1000 / - |
|  | Anti-Chicken / Alexa-647 | Jackson ImmunoResearch | 703-605-155 | Donkey polyclonal | 1:1000 / - |
|  | Anti-Rabbit / HRP | Invitrogen | 31466 | Goat polyclonal | - / 1:5000 |
|  | Anti-Chicken / HRP | ThermoFisher | A16054 | Goat polyclonal | - / 1:5000 |
| Dyes | DAPI | Sigma-Aldrich | D9542 | - | 1 µg/ml |
|  | AM4-64 | Biotium | 70025 | - | 10 µM |
| Quencher | SCAS (4-Sulfonato calix [8] arene, sodium salt) | Biotium | 70037 | - | 0.5 mM |

Figure S1: Confocal images of SH-SY5Y cells corresponding to the merged image of DPD 7 in figure 1. Synaptophysin (orange), PSD95 (green), DAPI (blue), β-tubulin III (red) and brightfield (grey). Scale bars: 20 µm. Sparse staining of synaptophysin and PSD95 shows beginning differentiation towards a neuronal phenotype. Neurites start to elongate and connect to neighboring cells.

Figure S2: Confocal images of SH-SY5Y cells corresponding to the merged image of DPD 14 in figure 1. Synaptophysin (orange), PSD95 (green), DAPI (blue), β-tubulin III (red) and brightfield (grey). Synaptophysin is seen in a more localized synaptic puncta manner, while PSD95 does not show a strong increase compared to DPD 7. Scale bars: 20 µm.

Figure S3: Confocal images of SH-SY5Y cells corresponding to the merged image of DPD 21 in figure 1. Synaptophysin (orange), PSD95 (green), DAPI (blue), β-tubulin III (red) and brightfield (grey). Synaptophysin stain increases upon longer differentiation, while PSD95 does not increase. β-tubulin III becomes more localized during maturation. Scale bars: 20 µm.

Figure S4: Confocal images of SH-SY5Y cells corresponding to the merged image of DPD 28 in figure 1. Synaptophysin (orange), PSD95 (green), DAPI (blue), β-tubulin III (red) and brightfield (grey). Presynaptic puncta become more abundant and slight post-synaptic puncta are present indicating further maturation. Axon bundles form and present in a more branching manner and appear more pronounced. Scale bars: 20 µm.

Figure S5: Representative confocal images of undifferentiated SH-SY5Y cells (DPD0) immunostained for synaptic and neuronal markers. Undifferentiated SH-SY5Y cells exhibit low-level expression of synaptic proteins without the punctate organization or polarized morphology characteristic of mature neuronal synapses. A: DAPI nuclear counterstain (blue). B: Synapsin shows weak and diffuse cytoplasmic staining without discrete puncta (cyan). C: PSD-95 signal is low and lacks organized postsynaptic-like clustering (green). D: VGLUT staining is sparse and discontinuous (yellow). E: GluA2 displays heterogeneous and predominantly non-punctate distribution (pink). F: Rab3A exhibits diffuse cytosolic localization without clear presynaptic enrichment (magenta). G: βIII-tubulin highlights limited and poorly organized neuritic processes (red). H: Synaptophysin signal is weak and non-clustered (orange). Scale bars: 50 µm.

Figure S6: Secondary antibody–only controls for immunofluorescence staining in differentiated (DPD28) SH-SY5Y cells. SH-SY5Y cells were processed using the full immunofluorescence staining protocol but incubated without primary antibodies, followed by incubation with the indicated species-specific secondary antibodies conjugated to Alexa Fluor dyes. Across all conditions, no specific fluorescence signal above background was detected. Nuclei were counterstained with DAPI (blue). A: Alexa Fluor 488–conjugated secondary antibodies raised against rabbit (green), B: Alexa Fluor 488–conjugated secondary antibodies raised against goat (green), C: Alexa Fluor 488–conjugated secondary antibodies raised against guinea pig (green), D: Alexa Fluor 564–conjugated secondary antibodies raised against rabbit (orange), E: Alexa Fluor 564–conjugated secondary antibodies raised against mouse (orange), and F: Alexa Fluor 648–conjugated secondary antibody raised against chicken (red). Scale bars: 50 µm.

Figure S7: iPSC-derived motor neurons and primary cortical neurons as positive controls for synaptic puncta organization. Representative confocal images of iPSC-derived motor neurons (A1–A3), and primary cortical neurons (B1–B3) immunostained for the presynaptic marker synaptophysin (SYP, orange) and the postsynaptic marker PSD-95 (green). Both neuronal systems exhibit extensive neurite networks with discrete, punctate localization of synaptic proteins along neurites. Merged images(A3, B3) reveal frequent apposition of pre- and postsynaptic markers, consistent with synapse-like structures. These neuronal cultures serve as positive controls, validating the detection of synaptic puncta under the applied staining and imaging conditions. Scale bars: 20 µm.

Figure S8: Electron micrographs of SH-SY5Y cells at A: DPD 7, D: DPD 14, B: DPD 21, and C: DPD 28 differentiated using RA + BDNF and E: DPD 21 and F: DPD 28 differentiated using NTFs. Neurites increase in length and number over differentiation period gradually with a slightly more bundled phenotype in cultures differentiated with RA + BDNF in comparison to the more separated neurite network observed upon addition of the NTFs CNTF, GDNF and IGF1. Scale bars: 5 µm.

Figure S9: Exemplary electron micrographs of SH-SY5Y cells differentiated with RA + BDNF and additionally Cholesterol A: Irregularly shaped synaptic-like vesicles in neurite extension lacking the characteristic synaptic structure of a postsynaptic counterpart. B: Axons with microtubules containing long tubular mitochondria, and LDCVs. Abbreviations: Mito: Mitochondria; MT: Microtubule; LDCV: large dense core vesicles; SVLV: Synaptic vesicle-like synaptic vesicles. Scale bars: 500 nm.

Figure S10: Cryo-EM Micrographs of neurite extensions in SH-SY5Y cells at DPD28. A: Densely packed neurite network of SH-SY5Y cells. LDCVs can be seen in neurites. B: Varicosity in a neurite bulging out to the edges of a holey carbon grid. Scale bars: 2 µm (A), 100 nm (B)

Figure S11: Cryo-EM micrograph (left) and cryo-ET (right) of multivesicular bodies (MVB) containing protein decorated vesicles, which - along with the lack of connectors and an active zone - clearly differentiates it from a presynaptic bouton. Abbreviations: Mito: mitochondria; ER: endoplasmatic reticulum; Lyso: lysosome; Ribo: ribosome. Scale bar: 200 nm.

Figure S12: Movement of fluorescent signal after AM4-64 staining. Selected time frames from the live staining experiment shown in Figure 5. SH-SY5Y cells at DPD 21 and DPD 28 were imaged under standard culture conditions or with additional NTFs. Fluorescence intensity is shown using the same pseudocolor scale as in Figure 5. Red and yellow arrows indicate two selected puncta tracked over time. The second frame was acquired directly after the addition of high-potassium Tyrode’s solution (+KCl) to stimulate exocytosis. Anterograde and retrograde movements of puncta without fluorescence loss, regardless of stimulation, suggest the absence of stimulus-evoked synaptic vesicle exocytosis. Puncta move out of focal plane (red arrow in DPD21), but do not undergo exocytosis. Corresponding live movies in Figure S16-19. Scale bars: 10 µm.

Figure S13: Confocal image of SH-SY5Y cells at DPD 28 differentiated with additional NTFs. Synaptophysin (orange), PSD95 (green), DAPI (blue), β-tubulin III (red) and brightfield (grey). Scale bars: 20 µm.

S14: Cryo-EM micrographs of thin projections in SH-SY5Y cells. Thin projections containing actin bundles are more frequent in early differentiation points and appear to be replaced with neurites containing microtubules upon further differentiation. A: SH-SY5Y cells at DPD 14 showing only few neurites that contain straight organized actin bundles characteristic of TNTs; A1: Zoom in of a possible TNT with actin structure and extracellular vesicles budding. A2: Zoom in of a branching extension with actin structure. B-D: thin TNT-like extensions and neurites at DPD 28. B: Overview of thin neurites of SH-SY5Y cells. C: TNT-like structures with bulging phenotype crossing each other. D: EVs budding in TNTs-like extensions.

***Movies***

S15: Movie of the tomogram seen in Figure 3A

S16: Movie of the tomogram seen in Figure 3B

S17: Movie of the tomogram seen in Figure 3C

S18: Movie of the tomogram seen in Figure 3D

S19-22: Movies of live-imaging seen in Figure 5 and S8

S23: Movie of the tomogram seen in Figure S8B
